# Physical Activity of University Students During COVID-19 Restrictions: Evidence from Poland

**DOI:** 10.3390/ijerph23060820

**Published:** 2026-06-20

**Authors:** Piotr Gabryjończyk, Anna Jęczmyk, Monika Wojcieszak-Zbierska, Jarosław Uglis, Jan Zawadka

**Affiliations:** 1 Institute of Economics and Finance, Warsaw University of Life Sciences—SGGW, 02-787 Warsaw, Poland; jan_zawadka@sggw.edu.pl; 2 Faculty of Economics, Poznań University of Life Sciences, 60-637 Poznań, Poland; anna.jeczmyk@up.poznan.pl (A.J.); monika.wojcieszak-zbierska@up.poznan.pl (M.W.-Z.); jaroslaw.uglis@up.poznan.pl (J.U.)

**Keywords:** physical activity, physical effort, Polish university students, pandemic, COVID-19

## Abstract

**Highlights:**

**Public health relevance—How does this work relate to a public health issue?**

- A total of 55.8% of surveyed students did not undertake systematic physical activity during the pandemic (59.2% of females vs. 48.4% of males).
- Among inactive respondents, the primary reported internal constraints were a lack of willingness and insufficient motivation.

**Public health significance—Why is this work of significance to public health?**

- Passive attitudes toward physical activity were linked by 25.1% of females and 21.8% of males to a lack of active role models and pre-existing lifestyle habits.
- The study identifies a significant trend of physical passivity among the younger generation in Poland, which may impact future public health outcomes.

**Public health implications—What are the key implications or messages for practitioners, policy makers and/or researchers in public health?**

- The primary external barrier identified by active students was limited access to sports facilities.
- A vast majority of active respondents adapted to restrictions by exercising at home or in the immediate vicinity of their residence.

**Abstract:**

This study aims to empirically analyze the patterns, intensity, and perceived barriers to physical activity among Polish university students during the COVID-19 pandemic. The research utilized a diagnostic survey method, employing a questionnaire. The online survey was conducted from December 2020 to May 2022 via the Webankieta.pl platform. The minimum sample size, calculated using the standard formula for estimating a proportion in a large population, was set at 1100 participants and was exceeded, with 1260 students providing valid responses. The results show that over half (55.8%, mainly women) of the respondents did not participate in regular physical activity during the pandemic. Participants cited lack of desire, fatigue, and low motivation—not pandemic restrictions—as primary reasons. Conversely, 44.2% of respondents, mostly men, reported engaging in regular physical activity. Most engaged in moderate-intensity activities two to five times a week, with vigorous activities performed slightly less often. Women were more likely to do both types, while men favored strength training. The most common activities included walking (61.6%), simple gymnastic exercises (43.1%), strength training with equipment (35.0%), cycling (34.5%), and calisthenics (30.2%). The majority (81.3%) exercised at home or nearby (33.4%). Reported barriers, especially among those who exercised regularly, were pandemic-related, such as limited or closed access to gyms, fitness centers, and pools (59.1%), along with time constraints (44.7%) and low motivation or determination (32.0%). The findings emphasize the importance of targeted interventions to boost physical activity among university students, particularly women and those with fewer financial resources. Universities should consider implementing programs that promote accessible, regular activity and initiatives to enhance motivation and foster long-term, health-promoting habits.

## 1. Introduction

Insufficient physical activity remains a critical public health challenge, directly impacting both physical and mental well-being while increasing the risk of chronic noncommunicable diseases [1,2]. These issues have been further exacerbated by the COVID-19 pandemic [3], which introduced structural barriers such as lockdowns, remote learning, and restricted access to sports facilities. Such restrictions have significantly disrupted the daily routines of young adults, a demographic already prone to sedentary behaviors [4].

While global studies have documented a general decline in physical activity levels during the pandemic [5], particularly among younger populations [6], there remains a relative scarcity of empirical data focused on the behavioral adaptations of higher education students in Poland [7]. Most existing literature addresses general health impacts [8,9], yet the specific responses of Polish students to prolonged external restrictions require further investigation [10].

Although the study was conducted during the COVID-19 pandemic, its relevance extends beyond the crisis context. The pandemic disrupted students’ existing patterns of physical activity, but it also made it possible to better distinguish between external barriers, such as sanitary restrictions and the closure of sports infrastructure, and more persistent determinants, such as lack of motivation, lack of habits, fatigue, time constraints, or financial situation [11,12,13,14,15]. Therefore, the findings may also be useful in the post-pandemic context, in which universities continue to face sedentary lifestyles, low motivation for physical activity, and unequal access to different forms of exercise.

This study helps address a knowledge gap regarding students’ physical activity. It shows which barriers were pandemic-related and which may persist after the restrictions have ended. The findings also indicate that previous exercise habits, gender, and students’ financial situation were relevant factors. Also forms of activity, such as home-based exercise, walking, and physical activity near the place of residence, were identified, which may be used in accessible and flexible university health promotion programs.

The aim of this study is to empirically analyze the patterns, intensity, and perceived barriers of physical activity among Polish university students during the COVID-19 pandemic, including comparisons between genders and between the profile of exercise practice. By identifying these behavioral patterns, the study provides evidence to support the development of targeted health promotion strategies in the post-pandemic academic environment. The results will allow us to understand not only the scale of the phenomenon, but also the specific barriers declared by students in Poland.

## 2. Literature Review

### 2.1. Definition and Meaning of Physical Activity

Physical activity is a fundamental determinant of health, influencing physical, emotional, and social development [16,17]. It is recognized as a sine qua non condition for maintaining optimum mobility and overall well-being across all age groups [18]. Research consistently demonstrates a positive correlation between physical activity levels and subjective health assessments [19].

The World Health Organization (WHO), after Caspersen et al., defines physical activity as any bodily movement produced by skeletal muscles that requires energy expenditure, encompassing exercise, active commuting, professional tasks, and leisure time activities [20]. Beyond individual choices, engagement in physical activity is shaped by social, economic, and environmental factors, including the availability of sports infrastructure and regional cultural traditions [18].

### 2.2. Physical Activity of Students

University students represent a unique social group whose lifestyle is shaped by academic schedules, financial resources, and the transition to independent decision-making [17,21,22]. Despite high health awareness, this population often faces barriers that make regular exercise difficult. Entering higher education is frequently associated with a decline in physical activity and a shift toward sedentary behavior—a trend that intensified during the COVID-19 pandemic [23,24].

Research conducted during the pandemic confirms significant disruptions in students’ health behaviors. For instance, studies by Aslan et al. and Pilecka et al. reported a substantial reduction in weekly activity minutes, even among students of physical education [24,25]. While a minority of students utilized the pandemic as an opportunity to maintain or improve fitness through home-based exercise, many experienced a loss of motivation, which was associated with a reported decline in their physical and mental health [26,27,28,29].

The relationship between physical activity, sleep quality, and mental health is well-documented; low activity levels are often linked to increased stress, anxiety, and psychosomatic disorders [30,31,32]. Factors such as gender, year of study, and academic specialization further differentiate these activity levels, underscoring the need for targeted interventions and hygiene education within the university environment [9,33,34].

### 2.3. Impact of COVID-19 on the Ability to Engage in Physical Activity

The COVID-19 pandemic introduced unprecedented global restrictions, including lockdowns, social distancing, and the closure of sports and recreation facilities [35]. These measures significantly reduced social mobility and disrupted traditional forms of active leisure [10,36,37]. The resulting isolation and increased sedentary time have been linked to a decline in psychological well-being, manifesting in heightened levels of anxiety, stress, and depression [26,38].

For the university population, the transition to online learning fundamentally altered daily routines [39]. The lack of access to organized sports and the suspension of traditional physical education classes led many students to significantly reduce or entirely discontinue their previous physical activity habits [40]. Consequently, the pandemic exacerbated the existing problem of physical inactivity, while a shift toward individual and home-based exercise patterns was observed in the students’ reports [41].

## 3. Materials and Methods

### 3.1. Data and Methods

The study relied on a questionnaire-based diagnostic survey with 19 closed and semi-closed questions. A convenience sample of students from several dozen Polish universities was recruited by the staff responsible for remote teaching upon the authors’ request. Participation was voluntary and anonymous, and students were informed about the purpose of the study and the use of their data before they started the survey. Completion and submission of the online questionnaire were considered as provisions of informed consent. The study was conducted from December 2020 to May 2022 and used the Webankieta.pl website.

The minimum sample size for the survey was determined using the standard formula for estimating a proportion in a large population:

$$n=\frac{Z^{2}\cdot p\cdot (1-p)}{e^{2}}$$

where *n* is the required sample size, *Z* is the z-score corresponding to the desired confidence level, *p* is the expected proportion, and *e* is the margin of error. Assuming a 95% confidence level (*Z* = 1.96), a conservative expected proportion of *p* = 0.50 and a margin of error of 3% (*e* = 0.03), the minimum required sample size was calculated to be approximately 1067 respondents. On this basis, the minimum sample size was set at 1100 participants, which was exceeded in the study (valid responses were obtained from 1260 students).

### 3.2. Variables

The primary dependent variable was engagement in physical activity, measured dichotomously (yes/no). Respondents were classified as physically active if they reported engaging in aerobic activity for at least 20 min, strength training of major muscle groups, or gymnastics/stretching at least twice a week during the last month.

Independent variables included gender (male/female), age (in years), type of study (full-time/part-time), place of residence (categorized by population size), and self-reported financial situation (5-point ordinal scale from very poor to very good). Additional variables included self-assessed physical fitness (5-point Likert scale) and reported barriers to physical activity (multiple choice items). The questionnaire was developed by the authors based on previous literature on physical activity and barriers among university students. To ensure clarity and content relevance, the instrument was pilot-tested among a group of 30 students representing the target population. Participants were asked to assess the clarity and comprehensibility of the items. Based on their feedback, minor wording adjustments were introduced. The pilot data were not included in the final analysis.

### 3.3. Statistical Analyses

Data collected during the study was subject to a statistical analysis with the use of STATISTICA 13.3. The Mann–Whitney U test and the chi square independence test were employed in order to assess the differences in the characteristics considered between female and male participants. The results are deemed significant at *p* < 0.05. Additionally, a multivariable logistic regression analysis was performed to assess independent associations between selected variables and physical activity. The dependent variable was engagement in physical activity (yes/no). The initial set of independent variables included gender, age, type of study, place of residence, and financial situation. Variables that did not meet model assumptions or showed insufficient variability were excluded from the final model. Specifically, type of study and place of residence did not meet the inclusion criteria and were therefore omitted from the final analysis. Odds ratios (ORs) with 95% confidence intervals (CIs) were calculated. A binomial logistic regression model was applied.

## 4. Results

The survey was administered to 1260 students of Polish tertiary education establishments from nearly 40 academic centers located in all 16 Polish voivodeships. Most of them were women (68.3%), and no one declared a gender other than male/female. Although the members of the sample differed in age (from 17 to 48 years), people aged up to 20 prevailed and the mean age was 21.7 (because the sample was composed of students). The vast majority of the respondents were students of public universities (over 90%) enrolled in full-time programs (nearly ¾ of the sample). Students of first-cycle programs formed the largest group (a total of 83%). Although the respondents included representatives of many fields of study, the largest groups were tourism and recreation and hospitality students as well as economy students (in both cases with a much greater share among women). Most respondents originated from a city (almost 60%) and lived in a city when the study was carried out (nearly 70%). Nearly 93% of the respondents were Poles (with Ukrainians and Belarusians being the other largest national groups). The respondents viewed their financial situation as quite satisfactory. Most of them (nearly 56%) believed it to be good or very good (slightly more frequently by men). Over one third considered it to be average. Table 1 presents the details for the sample as a whole and split by gender.

More than half of the respondents (55.8%) did not engage in physical activity during the pandemic. Women were less likely to engage in physical activity than men (59.2% vs. 48.4%). Note also that the group of respondents ranked their own physical fitness at 3.3 on a scale from 1 (extremely weak) to 5 (extremely good). Women ranked it at 3.2, whereas the rank given by men was slightly higher (3.5). A Mann–Whitney U test indicated a statistically significant difference between groups (U = 146396.5, *p* < 0.001), with a small effect size (r_rb_ = 0.15).

**Figure 1:**
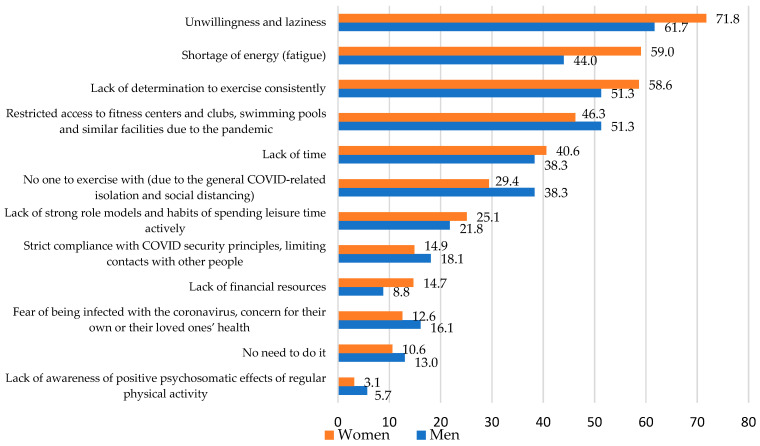
Reasons for not engaging in consistent physical activity during the COVID-19 pandemic among the respondents (%). The respondents could pick more than one answer, N = 703. Source: own study.

The reasons referred to by the respondents in justifying their lack of consistent physical activity during the pandemic include unwillingness, laziness, shortage of energy, fatigue and lack of determination (Figure 1). It was less common for them to indicate restrictions caused by the outbreak of the pandemic, i.e., restricted access to fitness centers and clubs, swimming pools and similar facilities, having no one to exercise with (because of the general COVID-related isolation and social distancing), and strict compliance with isolation principles. The latter were slightly more frequently cited by men.

To assess independent associations between selected variables and physical activity, a multivariable logistic regression analysis was performed. The results are presented in Table 2.

Gender was a significant predictor of physical activity. Male students were more likely to engage in physical activity compared to female students (OR = 1.54, 95% CI: 1.20–1.97, *p* = 0.001). The financial situation was also significantly associated with physical activity. An increase in the self-reported financial situation was associated with higher odds of engaging in physical activity (OR = 1.32, 95% CI: 1.12–1.56, *p* = 0.001). Age was not significantly associated with physical activity (OR = 1.03, 95% CI: 0.99–1.06, *p* = 0.108). Thus, gender and financial situation remained significant predictors of physical activity in the final model. In contrast, the variable “type of study” was not included in the final model due to insufficient variability in the dataset.

Further analyses revealed significant gender differences in the frequency of all examined types of physical activity, including moderate (χ^2^ = 54.79; *p* < 0.001; V = 0.21), intense (χ^2^ = 25.64; *p* = 0.001; V = 0.14), strength training (χ^2^ = 51.93; *p* < 0.001; V = 0.20), and simple gymnastics such as pilates or stretching (χ^2^ = 104.28; *p* < 0.001; V = 0.29). The respondents most often engaged in moderate-intensity activities, typically two to five times per week, while intense physical activity was also relatively common. Women slightly more frequently reported both moderate and intense activities. Strength training of large muscle groups (preferred more often by men) was usually performed two to three times a week (Table 3). Note also that physically active students rated their level of physical fitness much higher (3.8 on a scale of 1 to 5, with 3.8 for women and 3.9 for men) than those who did not engage in physical activity on a consistent basis. Simple gymnastics, such as pilates or stretching, were slightly less popular, but much more common in women.

The respondents largely differed in their preferred forms of physical activity during the pandemic. However, only five of them were consistently practiced by at least 30% of respondents (on average for both genders), namely: walking (61.6%) and simple gymnastics (43.1%, both much more preferred by women), strength training with equipment at home or in a fitness center (35.0%, much more often indicated by men), biking (34.5%) and calisthenics, i.e., strength training that utilizes an individual’s body weight (30.2%). For details, see Table 4. Detailed statistical results (χ^2^, *p*-values, and Cramér’s V) for gender differences in the mode of participation are provided in Supplementary Table S1.

Subsequent analyses showed significant gender differences in most forms of physical activity, with the strongest associations observed for dancing, aerobics, yoga, and group fitness activities.

Running (36.6%), swimming (32.7%), team sports (26.8%, significantly more for men: 33.0%), scooter riding, roller skating, skateboarding (26.0%, significantly more for women: 29.3%), as well as exercising at home with the use of an elliptical trainer, rowing machine, treadmill, etc. (25.0%) proved to be the most popular activities performed sporadically (i.e., indicated by ¼ of the respondents, except for those discussed above).

**Figure 2:**
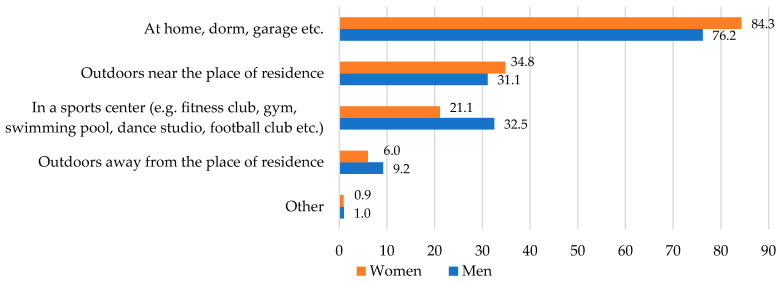
Places where the respondents performed physical activity during the pandemic (%). The respondents could pick more than one answer, N = 557. Source: own study.

During the pandemic, the vast majority of students exercised at home (81.3% in total, 84.3% of women and 76.2% of men) or in its immediate vicinity (33.4% in total, 34.8% of women and 31.1% of men). These results refer to respondents who reported engaging in physical activity (see Figure 2; N = 557).

A total of 25.3% of the respondents performed physical activity in different kinds of sports facilities. Men clearly dominated this group, which is likely because of them much more frequently choosing strength training (usually practiced in fitness centers).

**Figure 3:**
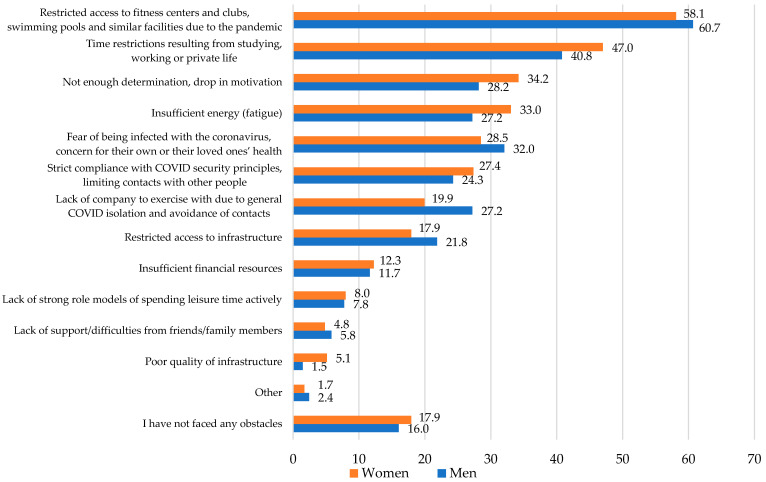
Obstacles and difficulties faced by respondents when engaging in consistent physical activity during the COVID-19 pandemic (%). The respondents could pick more than one answer, N = 557. Source: own study.

An important aspect of this study concerned the obstacles and restrictions faced by respondents when engaging in physical activity. The most frequently cited issues were related to the consequences of the pandemic, including limited or suspended access to fitness centers, clubs, swimming pools, and similar facilities (over 59.1%). Another commonly reported barrier was time constraints related to studying, work, or private life (nearly 44.7%, more often among women: 47.0%). In addition, lack of determination, decreased motivation to engage in physical activity, and fatigue were reported by 32.0% and 30.9% of respondents, respectively (in both cases, women predominated). These results refer to respondents who did not engage in consistent physical activity and are presented in Figure 3 (N = 557).

In addition to restricted access to sports and reaction facilities, the respondents also indicated other consequences of the pandemic which made it difficult for them to exercise on a regular basis. These were the fear of being infected with coronavirus, concern for their own or their loved ones’ health (29.8%, slightly more for men: 32.0%), strict compliance with COVID security principles, including limiting contacts with other people (26.2%, slightly more for women: 27.4%), and having no one to exercise with because of the general isolation and the intent to minimize contacts with other people (22.6%, much more for men: 27.2%). It needs to be emphasized that according to 7.9% of respondents, there is lack of generally available, widespread patterns of active leisure. A total of 17.2% of the respondents (slightly more for women: 17.9%) declared not to have encountered any obstacles or difficulties when engaging in physical activity during the pandemic.

In turn, there are huge noticeable differences between persons who exercised on a consistent basis and those who lacked consistency, i.e., who exercised sporadically or spontaneously (Table 5). This association was statistically significant among consistent exercisers (χ^2^ = 22.73, *p* < 0.001, V = 0.20), whereas no significant relationship was observed among non-consistent exercisers (χ^2^ = 4.83, *p* = 0.306, V = 0.08). Note however that both groups had a similar percentage (ca. ¼) of persons who did not make any essential change in their approach toward physical recreation during the pandemic. Another important remark is that in the group of regular exercisers, over 43% declared to have increased their activity in the pandemic. A drop in physical activity was reported by only 1/3 of representatives of that group. Conversely, a vast majority (almost 70%) of those who lacked consistency said that their physical activity dropped during the pandemic (an increase was reported by barely 7.5% of respondents in that group).

## 5. Discussion

The present study provides insight into physical activity patterns among Polish university students during the COVID-19 pandemic, highlighting both behavioral trends and factors associated with engagement in physical activity.

The post-pandemic relevance of the findings should also be emphasized. Although some barriers identified in the study were directly related to COVID-19 restrictions, many, such as lack of motivation, fatigue, poor exercise habits, time constraints, and financial limitations, may persist beyond the pandemic context. This suggests that physical inactivity among university students should not be interpreted only as a temporary consequence of sanitary restrictions, but also as a broader behavioral and public health issue.

The results indicate that more than half of the respondents did not engage in regular physical activity during the pandemic. This finding is consistent with previous studies conducted in various countries, including Slovenia, Italy, and Canada [42,43,44]. However, substantially higher levels of physical activity were reported in countries such as Malaysia and Indonesia [45], as well as among selected groups of students, including those enrolled in biomedical or health-related programs [28,46]. These discrepancies may reflect differences in study populations, cultural factors, or access to opportunities for physical activity.

A consistent finding across both the present study and prior research is the lower level of physical activity among female students compared to male students [47,48]. This pattern has been widely reported and may be related to differences in motivation, preferences for types of activity, and perceived barriers. In the current study, women more frequently indicated internal barriers such as lack of motivation, fatigue, and insufficient determination, whereas external constraints related to pandemic restrictions were less frequently reported.

Importantly, the multivariable logistic regression analysis demonstrated that gender and financial situation were independently associated with physical activity. Male students and those reporting a better financial situation were more likely to engage in physical activity, even after adjusting for other variables, whereas age was not a significant predictor. These findings suggest that both socio-demographic and economic factors play a role in shaping health-related behaviors during periods of restriction.

The association between financial situation and physical activity is consistent with previous research indicating that economic resources influence access to facilities, equipment, and opportunities for engaging in physical activity [49]. Even during periods of limited access to sports infrastructure, individuals with better financial resources may have been more able to adapt, for example by using home-based exercise equipment or digital training platforms.

The findings also suggest that pandemic-related restrictions were not the primary drivers of physical inactivity. Instead, internal factors such as lack of motivation and established habits played a more important role. This observation aligns with earlier studies indicating that barriers to physical activity among young adults are often stable over time and not limited to specific external circumstances [17,43,49].

The results obtained in this study are comparable to those reported by Lipošek et al., who found that 54.2% of Slovenian students engaged in physical activity at least two to three times per week [43]. Similar levels were observed among Italian students during the pandemic (44.7%) [42] and Canadian students, among whom 55.2% reported no physical activity [44]. However, earlier data from Central and Eastern Europe suggest that inactivity rates were considerably lower two decades ago [47], which may indicate a long-term decline in physical activity levels.

An important observation is the relatively low importance attributed to fear of COVID-19 infection as a barrier to physical activity. Instead, a substantial proportion of respondents indicated a lack of habits and role models for engaging in physical activity. This may reflect deficiencies in earlier stages of physical education and suggests the need for long-term behavioral interventions.

The study also revealed differences in preferred forms of physical activity. Most activities reported by respondents could be performed individually and did not require access to sports facilities, which reflects the conditions imposed by the pandemic. Similar patterns were observed in other studies, where home-based and outdoor activities such as strength training, jogging, and walking were the most common [50,51].

The analysis of changes in physical activity during the pandemic indicates that prior habits played a key role. Individuals who were physically active before the pandemic were more likely to maintain or increase their activity levels, whereas those who were previously inactive tended to reduce their activity further. Similar findings were reported in other studies [42,52,53].

These observations are supported by studies showing that individuals who engaged in home-based or outdoor activities before the pandemic experienced smaller declines in activity levels compared to those relying on organized or facility-based forms of exercise [53]. Gender differences were also observed, with women more likely to maintain or increase activity levels, while men more often reported a decline. Similar patterns have been reported in previous studies [28,54,55], although some contradictory findings also exist [56].

From a public health perspective, these findings highlight the importance of promoting physical activity through both structural and behavioral interventions. Universities should provide accessible and adaptable opportunities for physical activity, particularly under restrictive conditions, and support the development of long-term habits and motivation among students.

Several limitations of the study should be acknowledged. The use of a convenience sample limits the generalizability of the findings, and the reliance on self-reported data may introduce bias. In addition, the cross-sectional design does not allow for causal inference. The overrepresentation of female respondents (68.3%) should also be taken into account when interpreting the findings. This imbalance is not uncommon in survey-based research, as women are generally more likely to participate in voluntary questionnaires and are also more strongly represented in higher education populations. Consequently, some of the observed gender differences in physical activity levels and perceived barriers may be partially influenced by the composition of the sample.

Despite these limitations, the study provides valuable insights into physical activity behaviors during the COVID-19 pandemic and identifies key factors associated with engagement in physical activity among university students.

## 6. Conclusions

This study demonstrates clear differences in physical activity patterns among Polish university students during the COVID-19 pandemic. The pandemic and related restrictions affected many aspects of daily life, including the ability to engage in physical activity. The results of this study indicate that more than half of Polish university students did not engage in regular physical activity during the pandemic, with women being less active than men.

The findings of the multivariable logistic regression analysis showed that gender and financial situation were independently associated with physical activity. Male students and those reporting a better financial situation were more likely to engage in physical activity, whereas age was not a significant factor. These results suggest that both socio-demographic and economic factors play an important role in shaping physical activity behaviors among students.

Gender differences were also observed in the frequency and type of physical activity, as well as in preferred forms of exercise. Women more often engaged in moderate-intensity and individual activities, whereas men more frequently participated in strength training and higher-intensity forms of exercise. In addition, changes in physical activity during the pandemic were strongly associated with prior exercise habits, with previously active individuals being more likely to maintain or increase their activity levels.

The findings highlight the need for targeted interventions aimed at increasing physical activity among university students, particularly among women and individuals with lower financial resources. Universities should consider implementing programs that promote accessible and sustainable forms of physical activity, as well as initiatives focused on strengthening motivation and developing long-term health-related habits.

Our results are relevant not only for understanding students’ behavior during the COVID-19 pandemic, but also for planning post-pandemic health promotion activities targeting university students. The study indicates that physical inactivity among students cannot be explained solely by temporary restrictions, as motivational, habitual, economic, and gender-related factors also play important roles. Therefore, health promotion initiatives in the academic environment should combine access to sports infrastructure with activities that support motivation, regular habits, and accessible forms of physical activity, especially for students with limited financial resources and those without established exercise routines.

## Figures and Tables

**Table 1:** Characteristics of the respondents.

	Total N = 1260	Women N = 861	Men N = 399
**Age**			
Min	17	17	17
Max	48	48	47
Mean	21.7	21.6	21.9
Dominant	20	20	20
**University Type (%)**			
Public	90.5	89.6	92.5
Non-public	9.5	10.4	7.5
**Year of Study (%)**			
1st year of first-cycle studies	37.1	33.7	44.6
2nd year of first-cycle studies	26.0	27.8	22.3
3rd year of first-cycle studies	18.3	20.6	13.5
1st year of second-cycle studies	4.2	4.1	4.5
2nd year of second-cycle studies	8.0	8.5	7.0
Other	6.4	5.3	8.1
**Program Type (%)**			
Full-time	73.3	75.5	68.4
Extramural	26.7	24.5	31.6
**Field of Study (%)**			
Tourism and recreation; hospitality	38.1	42.7	28.1
Economics	34.6	36.0	31.6
Exact sciences	6.8	2.4	16.0
Technology and engineering	5.6	3.6	10.0
Agriculture, forestry, veterinary medicine	3.7	3.6	3.8
Natural sciences	3.1	3.4	2.5
Other	8.1	8.3	8.0
**Place of Origin (%)**			
Village	42.1	45.1	35.8
City with a population of up to 50,000	21.1	19.8	24.1
City with a population of 50,000–100,000	9.0	9.2	8.5
City with a population over 100,000	27.8	26.0	31.6
**Current place of Residence (%)**			
Village	30.3	30.0	31.1
City with a population of up to 50,000	15.1	13.7	18.0
City with a population of 50,000–100,000	7.1	7.7	5.8
City with a population over 100,000	47.5	48.6	45.1
**Nationality (%)**			
Polish	92.5	92.1	93.5
Other	7.5	7.9	6.5
**How do Individuals View Their Own Financial Situation (%)**			
Very good	12.1	12.4	11.5
Good	43.6	41.8	47.4
Average	36.6	38.1	33.3
Poor	6.9	7.0	6.8
Extremely poor	0.8	0.7	1.0

Source: own study.

**Table 2:** Multivariable logistic regression analysis of physical activity (N = 1260).

Variable	B	SE	Wald	OR (95% CI)	*p*
Gender (male vs. female)	0.432	0.130	10.98	1.54 (1.20–1.97)	**0.001**
Age (years)	0.029	0.018	2.58	1.03 (0.99–1.06)	0.108
Financial situation (per 1-point increase)	0.278	0.084	10.97	1.32 (1.12–1.56)	**0.001**

Note: B—regression coefficient; SE—standard error; OR—odds ratio; CI—confidence interval; *p*—significance level. Statistically significant results (*p* < 0.05) are shown in bold. Source: own study.

**Table 3:** Frequency of engaging in specific types of physical activity among the respondents (times per week) during the COVID-19 pandemic (%).

Activity Type	Gender	Number of Activities per Week									χ^2^	*p*	V
		0	1	2	3	4	5	6	7	More Than 7			
Moderate physical activity (30 min or more)	women	2.3	5.7	18.8	26.8	17.9	14.2	7.7	4.0	2.6	54.79	<0.001	0.21
	men	8.3	4.9	14.1	20.9	19.4	17.5	2.9	6.8	5.3			
Intense physical activity (20 min or more)	women	7.1	12.0	21.1	27.9	12.5	8.3	5.4	1.7	4.0	25.64	0.001	0.14
	men	7.8	12.6	18.4	20.4	12.6	12.6	4.4	4.4	6.8			
Strength training of large muscle groups	women	20.5	15.1	21.4	23.9	9.4	4.3	2.3	2.6	0.6	51.93	<0.001	0.20
	men	14.1	10.7	17.5	28.2	15.0	6.8	4.4	1.5	1.9			
Simple gymnastics, pilates, stretching	women	9.7	19.7	17.7	19.9	15.4	10.3	3.7	3.7	0.0	104.28	<0.001	0.29
	men	30.6	18.9	13.6	12.1	9.7	5.3	3.4	3.4	2.9			

Note: χ^2^—chi square independence test, *p*—significance level; V—Cramér’s V. Source: own study.

**Table 4:** The respondents’ preferred forms of physical activity during the COVID-19 pandemic (%).

Activity Type	Practiced on a Regular Basis (Unless the Season, Weather or COVID Restrictions Made It Impossible)		Practiced Sporadically		I Would Like to Try/Practice but I Am Unable to Do So for Various Reasons		Not Interested in It	
	W	M	W	M	W	M	W	M
Walking	71.2	45.1	25.1	40.8	2.3	3.9	1.4	10.2
Simple gymnastics, stretching, pilates	53.6	25.2	34.2	30.6	5.4	13.1	6.8	31.1
Strength training with equipment at home or in a fitness center	30.2	43.2	27.4	27.2	12.5	12.6	29.9	17.0
Biking	33.6	35.9	42.2	37.4	12.8	14.6	11.4	12.1
Calisthenics (training that utilizes an individual’s body weight)	29.1	32.0	32.5	31.6	11.1	15.5	27.4	20.9
Exercising at home with the use of an elliptical trainer, rowing machine, treadmill, etc.	22.5	16.5	25.9	23.3	16.8	14.6	34.8	45.6
Dancing	22.5	4.9	29.6	13.1	14.2	14.6	33.6	67.5
Running	14.0	18.4	35.6	38.3	21.4	16.5	29.1	26.7
Team sports (football, handball, volleyball, basketball)	6.8	25.7	23.1	33.0	16.5	15.0	53.6	26.2
Yoga	17.1	5.3	28.8	7.3	13.1	11.2	41.0	76.2
Aerobics	15.4	2.4	25.1	12.6	15.4	9.2	44.2	75.7
Downhill skiing and snowboard	7.4	12.1	14.8	11.7	17.1	19.4	60.7	56.8
Scooter riding, roller skating, skateboarding	8.5	5.3	29.3	20.4	14.2	10.2	47.9	64.1
Group exercising in a fitness club	9.1	3.9	13.7	6.8	21.9	9.7	55.3	79.6
Swimming	6.3	6.3	31.3	35.0	29.3	30.1	33.0	28.6
Ice skating (on indoor rinks)	6.3	5.8	25.9	12.1	15.4	10.7	52.4	71.4
Martial sports	2.8	9.7	5.4	9.2	22.2	22.8	69.5	58.3
Crossfit	5.1	4.4	16.5	12.6	16.8	12.6	61.5	70.4
Badminton	4.3	4.4	26.8	13.1	12.0	14.6	57.0	68.0
Nordic walking	4.3	3.9	11.7	3.9	10.8	6.3	73.2	85.9
Horse riding	4.3	3.9	6.6	3.9	19.9	9.7	69.2	82.5
Table tennis	2.0	6.8	12.8	21.4	13.1	16.0	72.1	55.8
Canoeing, rowing	2.3	5.8	13.7	12.6	14.8	15.5	69.2	66.0
Tennis	1.1	6.3	6.3	10.2	21.7	21.4	70.9	62.1
Cross-country skiing	2.0	4.4	3.4	4.4	12.3	8.7	82.3	82.5

Note: W—women; M—men. Source: own study.

**Table 5:** Changes in the respondents’ physical activity caused by the outbreak of the COVID-19 pandemic (%).

Changes in Individuals’ Physical Activity	Consistent Exercisers			Non-Consistent Exercisers		
	Total (N = 557)	Women (N = 351)	Men N = 206)	Total (N = 703)	Women (N = 510)	Men (N = 193)
Strong increase	20.5	22.8	16.5	1.1	1.2	1.0
Small increase	22.8	24.5	19.9	6.4	6.3	6.7
Same as before the COVID-19 pandemic	25.3	27.4	21.9	23.8	23.4	24.4
Slight drop	22.6	16.7	32.5	31.3	32.2	29.0
Strong drop	8.8	8.6	9.2	37.4	36.9	38.9

Note: Consistent exercisers: χ^2^ = 22.73, *p* < 0.001, V = 0.20; non-consistent exercisers: χ^2^ = 4.83, *p* = 0.306, V = 0.08. Source: own study.

## Data Availability

The original data presented in the study are openly available in OneDrive at https://sggwpl-my.sharepoint.com/:x:/g/personal/p616376_sggw_edu_pl/EXE6GovgtPJHlQM-haSnHtsBU3RVxg-vPy0bioOLJ5uwuw?CID=e9c062ff-06a7-4985-87af-af36c5c860f0&e=MamScx (accessed on 15 June 2026).

## Supplementary Materials

The following supporting information can be downloaded at: https://www.mdpi.com/article/10.3390/ijerph23060820/s1, Table S1: Association between gender and preferred forms of physical activity during the COVID-19 pandemic: χ^2^ tests and Cramér’s V.
