# Supplementary material for: Physical Activity of University Students During COVID-19 Restrictions: Evidence from Poland
Source: Int J Environ Res Public Health. 2026 Jun 20;23(6):820. doi: 10.3390/ijerph23060820 (PMC13300500; doi:10.3390/ijerph23060820)
Supplement: Supplementary file 1 [file ijerph-23-00820-s001.zip › ijerph-4237157-supplementary.pdf]

Supplementary Materials

**Table S1.** Association between gender and preferred forms of physical activity during the COVID-19 pandemic: $\chi^2$ tests and Cramér's V.

| Activity type | $\chi 2$ | *p* | V |
|---|---|---|---|
| Walking | 162.38 | <0.001 | 0.36 |
| Simple gymnastics, stretching, pilates | 198.72 | <0.001 | 0.40 |
| Strength training with equipment at home or in a fitness center | 46.18 | <0.001 | 0.19 |
| Biking | 7.83 | 0.050 | 0.08 |
| Calisthenics (training that utilizes an individual's body weight) | 12.63 | 0.006 | 0.10 |
| Exercising at home with the use of an elliptical trainer, rowing machine, treadmill etc. | 18.92 | <0.001 | 0.12 |
| Dancing | 412.47 | <0.001 | 0.57 |
| Running | 6.92 | 0.075 | 0.07 |
| Team sports (football, handball, volleyball, basketball) | 210.31 | <0.001 | 0.41 |
| Yoga | 365.18 | <0.001 | 0.54 |
| Aerobics | 421.63 | <0.001 | 0.58 |
| Downhill skiing and snowboard | 6.12 | 0.107 | 0.07 |
| Scooter riding, roller skating, skateboarding | 28.41 | <0.001 | 0.15 |
| Group exercising in a fitness club | 310.68 | <0.001 | 0.50 |
| Swimming | 3.92 | 0.270 | 0.06 |
| Ice skating (on indoor rinks) | 34.55 | <0.001 | 0.17 |
| Martial sports | 52.08 | <0.001 | 0.20 |
| Crossfit | 10.68 | 0.013 | 0.09 |
| Badminton | 30.47 | <0.001 | 0.16 |
| Nordic walking | 18.21 | <0.001 | 0.12 |
| Horse riding | 22.94 | <0.001 | 0.13 |
| Table tennis | 41.26 | <0.001 | 0.18 |
| Canoeing, rowing | 9.37 | 0.024 | 0.09 |
| Tennis | 19.84 | <0.001 | 0.13 |
| Cross-country skiing | 1.84 | 0.614 | 0.04 |

Note: $\chi 2$ **-** chi square independence test, *p* – significance level; *V* – Cramér's V.

Source: own study.
